# Cooling the soft tissue and its effect on perception of pain during infiltration and block anesthesia in children undergoing dental procedures: A comparative study

**DOI:** 10.15171/joddd.2019.025

**Published:** 2019-10-07

**Authors:** Sagorika Bose, Nishita Garg, Lumbini Pathivada, Ramakrishna Yeluri

**Affiliations:** ^1^Department of Pedodontics and Preventive Dentistry, Uttaranchal Dental College and Medical Research Institute, Dehradun, Uttaranchal, India; ^2^Department of Pedodontics and Preventive Dentistry, Teerthanker Mahaveer Dental College and Research Centre, Delhi Road, Moradabad – 244001, Uttar Pradesh, India

**Keywords:** Anesthesia, cooling, ice, injection, pain

## Abstract

***Background.*** This study assessed the effect of cooling the soft tissue site on the perception of pain in children undergoing local anesthesia for routine dental procedures.

***Methods.*** One hundred children, 6‒14 years of age, were assigned to either of the two study groups, i.e., group 1 (infiltration) and group 2 (block anesthesia). One side of the arch served as the test side, where an ice pretreatment (IP) of the soft tissue of the injection site was carried out using a tube of ice for one minute, whereas the opposite side served as the control, where no ice pretreatment (WIP) was carried out. This was followed by the gradual injection of local anesthetic solution. The children’s pain perception was assessed by VAS, WB-FPRS and SEM scales. The data were analyzed statistically.

***Results.*** WBS, VAS and SEM scores were significantly different between the WIP and IP in both groups, indicating that ice was effective in reducing the pain perception in children. Intergroup comparison revealed no significant differences (P>0.05), indicating that cooling was equally effective in infiltration and block anesthesia.

***Conclusion.*** Cooling the soft tissue site helped decrease pain perception during injection in children.

## Introduction

The traditional syringe still is the primary means of injecting local anesthesia; therefore, it is the focus of attention. Pediatric dentistry is all about managing pain effectively. There exists an ongoing search for ways by which the pain perceived during an injection can be minimized by producing a comfortable environment for children undergoing dental procedures. Although complete pain-free injection is almost impossible to achieve, various methods have been advocated to decrease the discomfort associated with intraoral injections.Some of these methods are the application of topical anesthetics,^1^ warming the anesthetic agents,^2^ reducing the rate of injection, distraction and other counter-irritation methods,^3^ buffering local anesthetic agent,^4^ etc. Other techniques that were in use include mechanical devices such as Computer Controlled Local Anesthesia Delivery System (CCLAD), Wand^5^ and Comfort Control Syringe (CCS)^6^ that regulate the flow rate and accelerate the speed of injection to minimize pain. Vibrotactile devices like Vibraject^7^ and Dental Vibe^8^ provide mechanical vibrations to the surrounding tissues and act as a counter stimulation. Needleless injector^9^ is a jet injection-based technology which creates a high-pressure blast of local anesthetic solution sprayed against the soft tissue, leading to penetration with minimal discomfort. However, these advanced techniques involve high cost, and the complex appearance of the equipment might further aggravate the child’s behavior. Despite the advancements in dentistry, to date, pain and anxiety continue to be a problem with injections.

Cooling the injured tissues has a long-standing history in medicine. The technique of local external cooling is being used for treating musculoskeletal pain, fractures, sports injuries, sprains, etc. Various studies have shown the benefits of postoperative and preoperative cooling therapy to decrease wound pain and edema.^10-12^ Limited studies are available on the role of topical cooling in dentistry. In 1989, Harbert^13^ observed decreased pain perception by the patients who underwent cooling in the palatal region before injection. Although the concept of cooling the soft tissue prior to injection procedures is established, the literature lacks such kind of application in orodental procedures, especially in children, with a few exceptions.^14-17^ Hence, this study was undertaken to assess whether the application of ice on the soft tissue site has any effect on the pain perceived by children undergoing injections. The objective was to introduce a cost-effective method which is less technique-sensitive but clinically very effective and can replace the traditional methods of inducing local anesthesia.

## Methods

The study protocol was approved by the ethics committee of Teerthanker Mahaveer Dental College and Research Centre, Moradabad, India. The parents or guardians of the selected subjects were provided with complete details of the study, who willingly allowed their children to participate in the study after signing the consent form. The sample size was calculated after power analysis which was 85% for this study. One hundred children aged six 6‒14, who met the inclusion criteria from 328 patients reporting to the Department of Pedodontics, were assigned to either group 1 or group 2.

### Inclusion Criteria

1. Patients requiring local anesthesia (infiltration/block) bilaterally on either the maxilla or mandible for various dental procedures
2. Cooperative patients (Frankel’s Class III or IV)
3. Healthy patients meeting the criteria of ASA physical status ‘I’

### Exclusion Criteria

1. Patients having significant behavioral problems
2. Patients with underlying systemic conditions
3. Children who were physically or mentally subnormal
4. History of a specific phobia or unpleasant experiences related to dental or medical settings
5. Patients allergic to anesthetic agents

**Group 1:** Fifty children requiring local infiltration anesthesia

**Group 2:** Fifty children requiring block anesthesia

**Figure 1:**
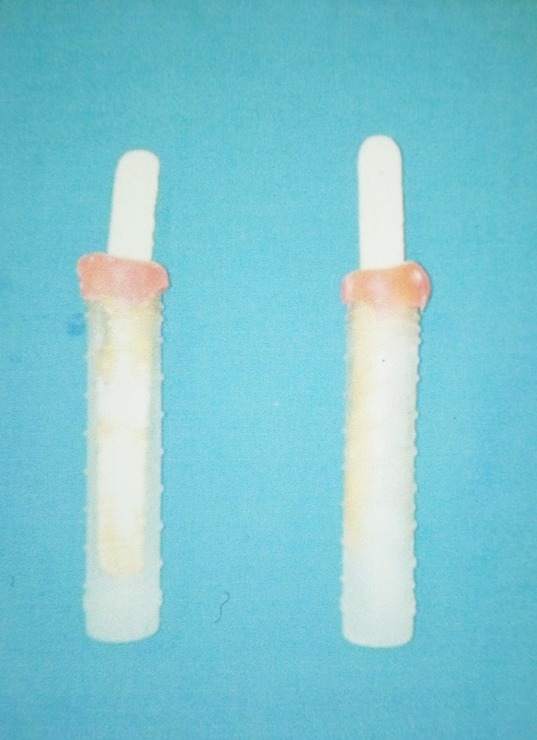
Preparation of the ice tubes.

Since each patient in both groups required local anesthesia administration bilaterally on either the maxilla or mandible, one side of the arch served as the test side (ice pretreatment, IP), whereas the opposite side served as the control (without ice pretreatment, WIP). The injection procedure was carried out on both sides on two different occasions. To determine whether the child would be treated for the test side or the control side on the first visit, randomization was carried out using the chit system.Each child was asked to pick a folded chit containing the term IP/WIP before the start of the procedure. The ice tubes were prepared by filling water in tube-shaped plastic containers (Figure 1). A wooden stick was inserted in each tube so that after freezing it could be held in hand and utilized for this study. The water-filled containers were then frozen at a temperature of -4ºC.

All the participating children were provided the necessary information regarding the injection procedure, familiarized with visual analog scale (VAS)^18^ and Wong Baker-Faces Pain Rating Scale (WB-FPRS)^19^ that were used in the assessment of pain. Behavior modification of the children was carried out using the tell-show-do method. A piece of sterile gauze was used to dry the soft tissue site prior to the intervention. For the test side, an ice pretreatmentof the soft tissue of the injection site was carried out using a tube of ice for one minute in the first visit. On the other hand, no ice pretreatment was carried out for the control side in the subsequent visit and vice-versa, based on the randomization protocol.The needle was then positioned on the appropriate site and slowly inserted. After successful negative aspiration, local anesthesia was administered gradually in 20‒30 seconds for infiltration and one minute in the case of the nerve block. The needle was withdrawn slowly after deposition of the solution.

All the participating children were assessed by VAS & WB-FPRS for the perception of pain during the injection procedure. VAS contains a 10-mm line from “0” indicating no pain to “10” indicating the worst possible pain. It intends to measure the pain intensity, and the children were asked to rate their pain on a scale of zero to ten. The WB-FPRS is an easy-to-use scale consisting of six drawn figures indicating a range from ‘no hurt’ to ‘hurts worst.’ Children were instructed to pinpoint a particular face that best described their pain perception. After the subjects’ self-reported measurements, their physical reactions were recorded by the operator using SEM^20^ (sound, eye, motor) scale during injections, which indicated the condition of each child ranging from ‘comfort’ to ‘severe discomfort’ on the basis of three variables, i.e., the child’s sounds (verbalizations), eye signs and body movements (Table 1).

**Table 1:** Parameters used in the SEM scale^20^

**Parameter**	**Comfort**	**Mild discomfort**	**Moderate discomfort**	**Severe discomfort**
Sound (S)	No sound	Non-specific sound (probable pain)	Verbal complaint, louder sound	Verbal complaint, shouting, crying
Eye (E)	No sign	Dilated eye without tear (anxiety sign)	Tears, sudden eye movements	Crying, tears all over the face
Motor (M)	Relaxed body and hand status	Muscular contraction, contraction of hands	Sudden body and hand movements	Hand movements for defense, turning the head to the opposite side

### Statistical Analysis

Statistical analysis of the data was carried out using SPSS 17 for Windows. Wilcoxon signed-rank test and Mann-Whitney test were used to compare pain perception during infiltration and block anesthesia with or without cooling the soft tissues. The significance level of all the statistical tests used in this study was pre-determined at P≤0.05.

## Results

Table 2 presents the comparison between IP and WIP in group 1 (Infiltration). The VAS, WBS and SEM scores were significantly higher in WIP as compared to IP, indicating the positive effect of cooling on pain perception during the infiltration technique. The differences observed between IP and WIP were found to be significant (P<0.05). Comparison of IP and WIP in group 2 (block anesthesia) is presented in Table 3. The VAS, WBS and SEM scores were also significantly higher in WIP as compared to IP, indicating a decrease in pain perception in the block anesthesia group. The differences observed between IP and WIP were also significant (P<0.05). The intergroup comparison between group 1 and group 2 for IP and WIP, respectively, are shown in Tables 4 and 5. No differences were observed, indicating that cooling was effective in infiltration and block anesthesia as well.

**Table 2:** Intragroup comparison of various pain rating scales with Ice Pre -treatment (IP) and without Ice Pre-treatment (WIP) in Group-1 (Infiltration) (Result of Wilcoxon signed rank test)

		**N**	**Mean rank**	**z value**	**p value**
**VAS_IP – VAS_WIP**	Negative Ranks	3	8.83	-5.495	<0.001***
	Positive Ranks	40	22.9		
	Ties	7			
	Total	50			
**WBS_IP – WBS_WIP**	Negative Ranks	1	13.50	-5.358	<0.001***
	Positive Ranks	37	19.66		
	Ties	12			
	Total	50			
**Sound_IP – Sound_WIP**	Negative Ranks	1	6.50	-3.000	.003**
	Positive Ranks	12	7.04		
	Ties	37			
	Total	50			
**Eye_IP – Eye_WIP**	Negative Ranks	0	.00	-5.745	.000***
	Positive Ranks	33	17.00		
	Ties	17			
	Total	50			
**Motor_IP – Motor_WIP**	Negative Ranks	0	.00	-2.236	.025*
	Positive Ranks	5	3.00		
	Ties	45			
	Total	50			

***very highly significant, ** highly significant, * significant

**Table 3:** Intragroup comparison of various pain rating scales with Ice Pre-treatment (IP) and without Ice Pre-treatment (WIP) in Group-2 (Block anesthesia) (Result of Wilcoxon signed rank test)

		**N**	**Mean rank**	**z value**	**p value**
**VAS_IP – VAS_WIP**	Negative Ranks	3	11.00	-4.974	<0.001***
	Positive Ranks	34	19.71		
	Ties	13			
	Total	50			
**WBS_IP – WBS_WIP**	Negative Ranks	4	16.50	-4.774	<0.001***
	Positive Ranks	34	19.85		
	Ties	12			
	Total	50			
**Sound_IP – Sound_WIP**	Negative Ranks	2	6.50	-2.841	.005**
	Positive Ranks	13	8.23		
	Ties	35			
	Total	50			
**Eye_IP – Eye_WIP**	Negative Ranks	2	16.50	-4.950	<0.001***
	Positive Ranks	30	16.50		
	Ties	18			
	Total	50			
**Motor_IP – Motor_WIP**	Negative Ranks	1	4.50	-2.121	.034*
	Positive Ranks	7	4.50		
	Ties	42			
	Total	50			

***very highly significant, ** highly significant, * significant

**Table 4:** Intergroup comparison of various pain rating scales with Ice Pre-treatment (IP) between Group-1 and Group-2 (Result of Mann Whitney test)

		**N**	**Mean rank**	**Mann-Whitney value**	**Wilcoxon value**	**p value**
**VAS_IP**	Infiltration	50	49.93	1222	2496	.839 NS
	Block anesthesia	50	51.07			
	Total	100				
**WBS_IP**	Infiltration	50	49.92	1221	2496	.833 NS
	Block anesthesia	50	51.08			
	Total	100				
**Sound_IP**	Infiltration	50	50.74	1238	2513	.897 NS
	Block anesthesia	50	50.26			
	Total	100				
**Eye_IP**	Infiltration	50	46.02	1026	2301	.073 NS
	Block anesthesia	50	54.98			
	Total	100				
**Motor_IP**	Infiltration	50	50.50	1250	2525	1.000 NS
	Block anesthesia	50	50.50			
	Total	100				

NS- Not Significant

**Table 5:** Intergroup comparison of various pain rating scales without ice pre-treatment (WIP) between group 1 and group 2 (the results of Mann-Whitney test)

		**N**	**Mean rank**	**Mann-Whitney value**	**Wilcoxon value**	**p value**
**VAS_WIP**	Infiltration	50	54.13	1068.500	2343.500	.194 NS
	Block anesthesia	50	46.87			
	Total	100				
**WBS_WIP**	Infiltration	50	54.30	1060.000	2335.000	.167 NS
	Block anesthesia	50	46.70			
	Total	100				
**Sound_WIP**	Infiltration	50	51.23	1213.500	2488.500	.766 NS
	Block anesthesia	50	49.77			
	Total	100				
**Eye_WIP**	Infiltration	50	49.22	1.18603	2.46103	.534 NS
	Block anesthesia	50	51.78			
	Total	100				
**Motor_WIP**	Infiltration	50	50.42	1246.000	2521.000	.965 NS
	Block anesthesia	50	50.58			
	Total	100				

NS- Not Significant

## Discussion

Elimination of pain in pediatric dentistry is an important aspect, especially during injections. One such method of elimination is the technique of cooling, which is also known as cryoanesthesia, i.e., blocking local neural transmission of painful stimulus by cooling a localized area. It can be delivered using ice or a refrigerant spray. Applying ice before or after painful procedures has been practiced for thousands of years and has been one of the first techniques for local anesthesia and analgesia. Ethyl chloride is also an excellent cooling agent that is in practice for controlling pain in various situations.^21,22^

Few reports^10-12^ are available in the literature on the use of cooling to assess the pain reactions caused by the local anesthetic injections. They all have reported significant results, indicating that pre-cooling was effective in alleviating pain associated with injections. As a matter of fact, there is little published data on the effect of cooling the injection site in dental procedures. Harbert^13^ presented the idea of pre-cooling technique for palatal injection and observed that prior palatal cooling is efficient in relieving injection prick pain. Similar findings were reported by Duncan et al^23^ after applying a cotton pellet saturated withdichlorodifluoromethane spray for 5 seconds prior to administrating palatal injections. Their results revealed less discomfort during needle penetration. Kosaraju et al^24^ compared the 5-second application of a refrigerant spray with the 2-minute application of a topical gel before local anesthetic injection in the posterior palatal site with a 30-gauge needle. They found that the refrigerant agent prior to anesthetic injection was more effective than the topical gel.

However, other chemical cooling agents are not as safe as ice. There can be hazards of frostbite or contact dermatitis on continuous exposure to a coolant spray. Ice presents an effective non-pharmacological and reliable way of pain management. Ice has been used as a therapeutic agent in the field of medicine for postoperative reduction in wound pain, sprains, fractures, sports injuries, burn cases, etc. Ice can be prepared and used in any form, including cubed ice or crushed pieces of ice. With these benefits, ice can easily be used for the technique of pre-cooling.

Considering the above facts and the inadequacy of such applications in children, the present study was undertaken. An age group of 6‒14 years was considered for this study since young preschool-aged children might become restless with the application of ice. Also, preschool children cannot be counted on to provide reliable and valid reports of their pain and distress. Only cooperative children were included in the study as pain is highly related to the experience of anxiety, and anxious children before injection tend to show more pain experience than non-anxious children. Various factors that might produce discomfort by ice in children are contact time of ice and the individual’s pain threshold level. Gadheri et al^15^ suggested 2‒5 minutes of application time for ice. The suggested time of ice application is nearly 2‒5 minutes. Adults might tolerate five minutes of application, and this might be a matter of concern in children because of behavioral issues. Due to these facts, we applied ice for only one minute in this study.Williamson et al^25^ found VAS as a valid, reliable and appropriate scale in the clinical practice. WBS was found to be a constructive self-report measurement of pain as it showed good validity. However, drawbacks of this scale might be a misjudgment of these different faces as sadness compared to pain. Various authors recommend the use of two types of pain assessment scales in clinical practice. The subjective assessment was made using SEM scale^20^ as it is a direct measure of the child’s body movements and vocalization.

This study showed that one-minute application of ice prior to local anesthetic administration reduced the pain perception significantly during the injection procedure. Infiltration and block anesthesia groups showed statistically significant differences (P<0.05). Significantly higher scores of VAS, WBS and SEM were obtained in WIP than IP, indicating the positive effect of ice on the pain perception in children. Pre-cooling was effective in reducing pain, irrespective of the kind of the local anesthesia administered due to the absence of differences between the groups. The results are also consistent with Aminabadi et al^14^ and Ghaderi et al,^15^ who reported similar results. Mohiuddin et al^16^ compared ice and local anesthetic gel before injection for the extraction of primary maxillary anterior teeth and found ice to be very effective. Lathwal et al^17^ reported that ice is definitely superior and has higher efficacy compared to benzocaine and refrigerant sprays during intraoral injections.

Wiswall et al^26^ surveyed the pain response to three different site preparations (pressure, pressure + topical anesthetic [20% benzocaine], and pressure + pre-cooling) prior to the greater palatine nerve block. They reported no significant VAS differences between the test groups and concluded that all of them were almost equally effective. The course of action of cooling in the reduction of pain can be explained by numerous theories. Cold stimulates myelinated ‘A’ fibers and activates pain pathways of inhibition, and thus the pain threshold is raised.^27^ In the present study, cylindrical tubes of ice were prepared and used for the technique of pre-cooling. The advantages of this technique are that it is comfortable, safe and physiologically effective. Also, ice is inexpensive and readily available everywhere. It is a material which is familiar to the patients; therefore, it is less likely to induce anxiety and subjective fear, especially in children.

Blinding the subjects and the evaluator was not possible due to the sensation of cold upon pre-cooling, and we consider it as a limitation. The temporary anesthesia produced by ice is of very short duration; therefore, the injection procedure had to be carried out very rapidly. Also, as pain is affected by a wide variety of contextual variables, the perception of pain varied from child to child, and the anticipated discomfort could be a critical factor in obtaining the results. The rate at which the anesthetic solution is deposited and the location of the injection site are other factors that could vary with each appointment. Prior experience of children with injections also plays a critical role in reaction to pain stimulus. Those subjects who were calm and readily accepted their first injection also reacted positively to the local anesthesia during the second visit, and vice versa. The reliability of the results could have been further improved by videotaping the injection procedure and allowing a third investigator to evaluate it. Keeping various advantages of local anesthesia in mind, this study was an effort towards delivering painless injections to reduce pain, increase patient compliance and improve the quality of care, and all the clinicians should be made familiar with this strategy.

## Conclusion

Cooling the soft tissue site significantly decreased the perception of pain (both infiltration and block anesthesia) in children during routine dental procedures. It proved to be an easy, reliable and cost-effective method of local anesthetic administration.

## Conflict of Interests

The authors declare no conflict(s) of interest related to the publication of this work.

## Authors’ contributions

SB: Contributed to the design, implementation of research, analysis of the results and writing of the manuscript. NG: Contributed to the design of the research, analysis and interpretation of the results and critical evaluation and editing of the manuscript. LP: Contributed to analysis and interpretation of the results and critical evaluation of the manuscript. RY Contributed to the design of the research, analysis and interpretation of the results and critical evaluation and editing of the manuscript.

## Acknowledgment

None.

## Funding

Self-funded.

## Ethics approval

The authors state that the Institutional Ethics Committee of Teerthanker Mahaveer Dental College & Research Centre affiliated to Teerthanker Mahaveer University, Moradabad, India has approved the design of the research and informed written consent was obtained from patient’s parent/guardian before the beginning of this study.
